# Pain, Anxiety, and Depression in Hemodialysis Patients Undergoing Different Types of Vascular Access: A Cross‐Sectional Study

**DOI:** 10.1155/da/5100502

**Published:** 2026-08-18

**Authors:** Leen Nasser, Tarek Youssef, Rama Saad, Fayez Al-Houssami Al-Jobeily, Fatima Zayyat, Ghida Chaaban, Montaser Taleb, Reem El Hajj Chehade, Youssef Khrayzat, Madiha A. Hassan

**Affiliations:** ^1^ Faculty of Medicine at Beirut Arab University, Beirut, Lebanon, bau.edu.lb

## Abstract

**Background:**

Chronic hemodialysis (HD) patients experience various psychological burdens including pain, anxiety, and depression which are often under‐recognized in routine clinical care. While the vascular access (VA) modality is a crucial factor in dialysis adequacy and patient outcomes, its participation in the psychosocial distress remains insufficiently assessed.

**Objective:**

To assess the association between the VA types, arteriovenous fistula and central venous catheter (CVC), and the prevalence of pain, anxiety, and depression in chronic HD patients.

**Methods:**

This multicentered cross‐sectional study included 415 adult patients undergoing HD for at least 1 year in six Lebanese hospitals. Participants with a documented diagnosis of psychological disorders or receiving antidepressants prior to dialysis initiation were excluded. Anxiety and depression were assessed through Hospital Anxiety and Depression Scale (HADS) while pain was evaluated using Numeric Rating Scale and Short‐Form McGill Pain Questionnaire.

**Results:**

The incidence of symptomatic anxiety was higher with arteriovenous fistula (75%) than with CVC (25%). Similarly, detection of depressive symptoms was higher with arteriovenous fistula (81%) than with CVC (19%). Some degree of pain has been reported by 54.5% of studied patients. Statistically, there was no significant difference between the type of VA and the psychosocial factors mentioned (*p*  > 0.05). In contrast, higher pain score and psychosocial distress were associated with dialysis and VA related complications (*p* = 0.018).

**Conclusion:**

These findings highlight the importance of routine systematic symptom assessment, complications management and prevention, and the integration of a multidisciplinary support team in HD care.

## 1. Introduction

Renal replacement therapy (RRT) is the preferred therapy for end‐stage renal disease (ESRD) patients who are categorized as stage 5 with eGFR < 15 mL/min/1.7 m^2^ until a renal transplant is available [1]. Most patients who started dialysis at an appropriate time showed a better life span [2]. Renal transplantation is a long‐term management that is governed by the availability of a compatible donor.

There are three main types of dialyses to apply according to their suitability to the patient’s clinical condition: hemodialysis (HD), peritoneal dialysis, and hemofiltration [3].

HD is the most common type of RRT that serves as an artificial kidney in patients who have lost all intrinsic kidney functions [3]. It can serve as an emergency solution for patients who have acute kidney injury, as well as a maintenance option for ESRD patients or those waiting for renal transplantation.

HD works by filtering the blood from excess water, toxins, and solutes through a semi‐permeable membrane in a dialyzer machine that separates solutes down the concentration gradient from the blood to the dialysate. The dialysate consists of electrolytes, purified water, and dextrose while lacking low‐molecular‐weight products found in uremic blood. This process of diffusion continues until the concentration of permeable waste products from both the blood and dialysate reaches equilibrium [3].

HD is done via a vascular access (VA) that allows the connection of the patient’s circulation to the machine. The main types of VAs are arterial venous fistula (AVF) and central venous catheter (CVC) [4]. An AVF is a connection between an artery and a vein done surgically, often in the upper arm or forearm, to allow the arterial blood to empty into the vein directly. This causes the vein to enlarge and withstand the needle insertions required in the procedures. A CVC is a flexible catheter inserted into a large central vein, most commonly the jugular vein, subclavian vein, or femoral vein. It is typically used for urgent cases that require immediate dialysis, for those who are waiting for an AVF to mature, or it can be a permanent VA in patients who do not have another option [4].

Some studies have addressed the prevalence of anxiety and depression among HD patients and found them to be higher than the general population [5] and especially in the age group >65 years [6].

HD aims to maintain physiological health and prolong patients’ survival rates. However, the psychosocial aspects of the patients are also crucial in maintaining their overall well‐being.

According to the World Health Organization, “health is a state of complete physical, mental, and social well‐being, not merely the absence of disease or infirmity” [7]. This highlights the importance of integrating emotional and psychosocial well‐being alongside physical treatment.

Pain, depression, and anxiety are some of the overlooked comorbidities that burden patients undergoing HD. They should not be considered secondary concerns since they directly impact patients’ quality of life, recovery journey, and adherence to treatment.

Pain is the most reported symptom among patients undergoing HD, with several studies reporting high prevalence rates [8–11]. This complaint hinders the patients’ daily activities and compromises their quality of life. Chronic pain usually assumes various forms, including generalized musculoskeletal, abdominal, or neuropathic pain. However, chronic pain is rarely assessed in routine clinical checkups of HD patients. It is overlooked to the extent where it has to be considered a part of the assessment of the HD process with special attention that should be paid to the VA‐associated pain. Chronic pain is usually associated with HD sessions; however, its effects can extend even further to affect patients between dialysis visits, treatment tolerance, and their overall mental state [8]. Persistence of pain and deterioration of mental health are both mutually related, and they can ultimately lead to feeling of depression and/or anxiety.

Masià‐Plana et al. [12] found that pain levels were generally low in HD patients, but authors did not examine the relation between pain, anxiety, depression, and the type of VA. To our knowledge, no study has been done in Lebanon that investigates pain in HD patients with different VA or its correlation to psychological distress.

As defined by the Diagnostic and Statistical Manual of Mental Disorders—Fifth edition Text Revision (DSM‐5‐TR), depression is the presence of a sad, empty, or irritable mood, accompanied by related changes that significantly affect the individual’s capacity to function [13]. Anxiety is defined as excessive worry and apprehensive expectation of a tragedy occurring more days than not for at least 6 months. Depression is usually accompanied by anxiety, often having interrelated telltales. Common signs may also include lassitude, sleep disturbances, and change in weight and appetite. The prevalence of depression and anxiety among HD patients has been reported to be around 69% to 37%, respectively [14]. This issue is also insufficiently addressed in dialysis care.

Furthermore, the clinical picture extends to affect the patient’s emotional state, treatment effectiveness, and adherence. Patients don’t only suffer the pathological effect of kidney failure but are also deprived of their family role, social status, occupation, motility, and time. They are bound to a monotonous lifestyle of stagnancy and pain, which will inevitably reflect their mental health state [15]. Another possible contributor to the spiraling mental state is suffering from any drawbacks linked to the type of VA. Although CVC is an urgent and acute treatment, it causes infections, discomfort, and limited mobility. Meanwhile, AVF is more stable, but it is still associated with pain due to repetitive needle pricks and body image concerns at the VA site. Many patients may avoid their social gatherings to avoid inquiry from others about the AVF. Semaan et al. [5] studied the prevalence of anxiety and depression in HD patients in a single center in Lebanon and found that both factors were relatively high and significantly correlated to each other.

While some studies discussed the impact of pain, anxiety, and depression on HD, few have delved into their relationship with the type of VA. Several studies reported that patients with CVC have higher anxiety and/or depression levels than those with AVF [16–18]. However, the small sample size of the studied population in these studies was not strong evidence in determining the relation between psychological distress and the type of VA. Further studies on larger samples will be of value in the assessment of the psychological aspect during the patient’s HD journey. Based on this, the current study aims to evaluate pain, anxiety, and depression in different VA types in hopes of building more holistic, patient‐centered treatment strategies and promoting individualized and emotionally focused dialysis care. We hope to help bridge the gap concerning the ambiguity of the difference in VA and its physiological and psychological correspondents.

## 2. Methodology

### 2.1. Study Design

This multicenter study used a cross‐sectional descriptive design across six dialysis centers in different regions in Lebanon. This design aims to assess and associate the prevalence of pain, anxiety, and depression in HD patients with different VAs. Data collection was done by collecting surveys, and no further clinical interventions were performed.

### 2.2. Sampling Participants

According to the Ministry of Public Health in Lebanon, the estimated population count of HD patients is 3307 distributed across six governorates, from which the sample size was calculated using Cochran’s formula. A confidence level of 95% and a margin of error of 5% were adopted for the calculation of the sample size, with a prevalence of 50%, as well as applying the finite population correction. The initially adjusted sample size was 345. After accounting for an anticipated 10% attrition rate, the final required sample size was 384 participants.

**Figure 1:**
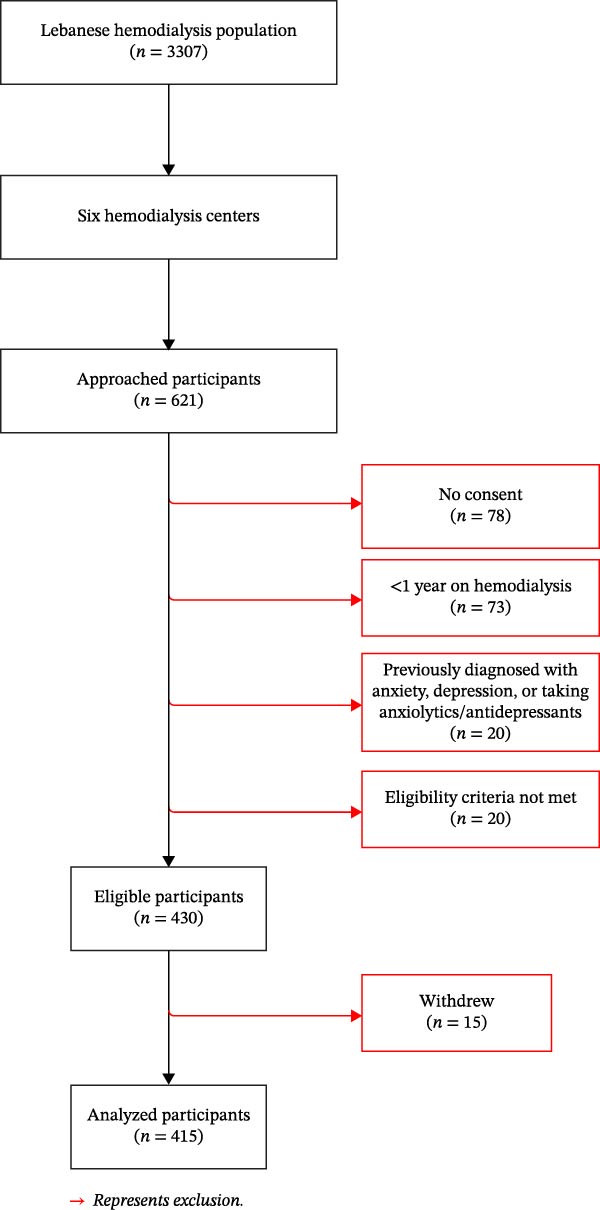
Participants recruitment and selection process according to the inclusion and exclusion criteria.

In total, 621 participants were approached, 415 of whom consented to enroll in the study and fit the inclusion criteria. Inclusion criteria consisted of Lebanese adults above 18 years old who have ESRD and permanent VA requiring lifelong HD. Participants were excluded if they were on HD for less than a year, previously diagnosed with anxiety/depression, or were on anxiolytics/anti‐depressants prior to their HD. Restricting the inclusion to those who have been on dialysis for more than a year was done to minimize the influence of the initial adjustment period following dialysis initiation. Other studies have adopted similar eligibility criteria in the selection process of their participants [19]. Furthermore, this criterion was chosen after several studies observed that time‐varying analysis was a crucial factor influencing the psychosocial status of dialysis patients [20, 21]. Hence, the 1 year cut‐off was selected to ensure the inclusion of patients with established dialysis experience and to reduce potential variability associated with recent dialysis initiation. Detailed description of the participants is shown in Figure 1. The hospitals were sampled by convenience, and data collection commenced after receiving the IRB approval. Participants were sampled by convenience, as well, per their oral and written consent.

### 2.3. Setting

**Figure 2:**
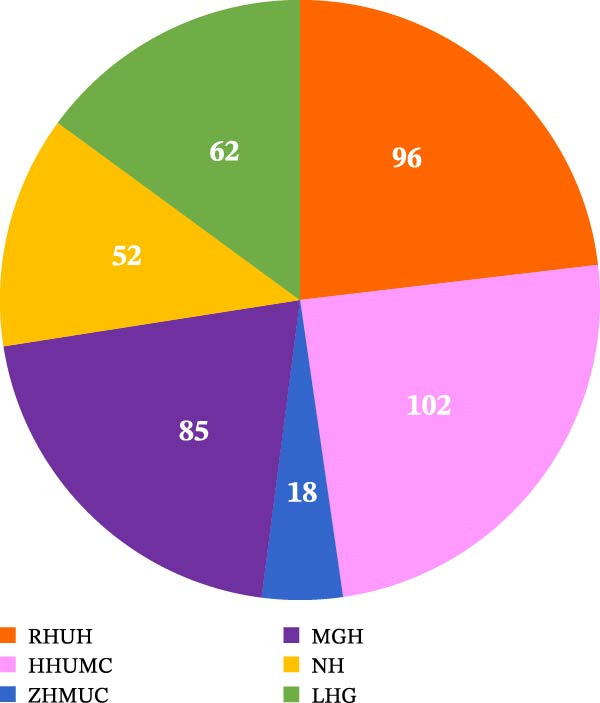
Number of participants collected from each hospital.

The study was conducted at six different dialysis centers across Lebanon. The data were collected during the beginning of July 2025 through August 2025 from Makassed General Hospital (MGH), Rafic Hariri University Hospital (RHUH), Lebanese Hospital Geitaoui (LHG), Zahraa Hospital University Medical Center (ZHUMC), Hammoud Hospital University Medical Center (HHUMC), and Nini Hospital (NH). The distribution of collected participants from each hospital is represented in Figure 2.

### 2.4. Data Collection Instruments and Procedure

The data collected constituted four main sections: a sociodemographic survey and three separate scales. The survey consisted of sociodemographic, dialysis history, and VA‐related questions. The survey was written in English and then back‐to‐back translated into Arabic and back to English. After that, both options were presented to the patients to choose their preferred language.

The first scale used was the Hospital Anxiety and Depression Scale (HADS), which assessed the patients’ psychological state. The other two scales, the Numerical Rating Scale (NRS) and the Short Form McGill Pain Questionnaire‐2 (SF‐MPQ‐2), were used to measure pain related to the patients’ dialysis and VAs. These scales were selected based on their relevance to our population, the number of questions each contained, the dual benefit that HADS can assess both anxiety and depression, and the availability of validated translated versions of each scale in Arabic. The NRS was added to measure the total pain perception of the participants, taking into consideration that the SF‐MPQ‐2 lacks this section. Several other studies utilized this scale or ones similar to it such as the visual analog scale (VAS) for the same purpose [22].

#### 2.4.1. HADS

The HADS scale consists of 14 questions divided into two subscales; a subscale for anxiety (HADS‐A) formed of seven questions and a subscale for depression (HADS‐D) formed of another seven questions. Each question has a score ranging from 0 to 3, and each subscale has a total score ranging from 0 to 21 [23]. The cutoff score for HADS‐A to recognize a patient with anxiety was chosen to be 6, and that for HADS‐D to recognize a patient with depression was chosen to be 7. This was decided according to a study done by Preljevic et al. [24],which argued that in patients with chronic diseases such as ESRD, the cutoff scores for HADS‐A should be lower as it has a low sensitivity at the recommended score. This allows the scale to have a better assessment of patient anxiety. The scales for HADS‐A and HADS‐D have a Cronbach’s alpha of 0.832 and 0.823, respectively. A value >0.8 is a good consideration of the scale’s reliability to be used in the study. The Arabic version of the scale was adopted from Terkawi et al. [25]. The scales were valid in both English and Arabic for our population.

#### 2.4.2. NRS

The NRS is a one‐question scale that identifies overall pain intensity. It has a score ranging from 0 to 10, where 0 indicates no pain, and 10 indicates the worst pain possible.

#### 2.4.3. SF‐MPQ‐2

The SF‐MPQ‐2 scale is formed of 22 questions divided into the following subscales: continuous pain (six questions), intermittent pain (six questions), neuropathic pain (six questions), and affective descriptors (four questions). Cronbach’s alpha for the subscales was calculated with the scores of 0.758, 0.804, 0.697, and 0.741, respectively. Each of the questions has a score from 0–10, where 0 indicates no pain and 10 indicates the worst pain possible, and the score for each individual subscale is calculated by finding the mean score of the questions in that subscale [26]. The Arabic version of the scale was adopted from Farhat et al. [22].

### 2.5. Data Collection

HD patients were recruited by medical students during their assigned dialysis shifts. All surveys were filled out as an online form on electronic devices by various team members over a 20 min interview with each patient. After thoroughly explaining the topic and terms of the study, informed consent was taken orally and electronically from each patient prior to initiating the interview. Team members informed the patients that their identities remain anonymous, and they had the right to withdraw at any moment throughout the interview. The entire survey was available in Arabic and English to accommodate different preferences.

### 2.6. Data Analysis

Microsoft Excel was used for cleaning, coding, and organizing the data. IBM SPSS version 23 was used for data analysis. Adjusting the sample at a confidence interval of 95%, a *p*‐value < 0.05 was considered significant. All categories were considered abnormally distributed per Kolmogorov–Smirnov (K–S) test as well as evaluating *Q*–*Q* plots and histogram distributions; hence, all values are reported as median (IQR Q1–Q3). For data analysis, the following tests were used: chi‐square, Mann Whitney *U*, Kruskal–Wallis, ANOVA, and Spearman rho for continuous variables’ correlation.

### 2.7. Ethical Consideration

Prior to data collection, approval from the IRB committee of each individual hospital was acquired. Ethical considerations were reviewed by their boards as well as our team members prior to any interaction to ensure that they were properly met. Bearing in mind that HD patients can be considered a vulnerable population, they were guided through the questionnaire by our team members, answering and clarifying any questions they had. The written and oral consents explained the study, the interview process, as well as its benefits and the lack of any physical or financial risks. All participants were informed that they have the right to decline or withdraw at any moment of the process without further consequences.

## 3. Results

### 3.1. Participants’ Description

A total of 415 participants were analyzed. The median age of the participants was 62 (52−71), and the majority were males (59.5%). Most of the participants were unemployed/retired (77.6%), and more than half resided in Beirut (53.5%). Smoking was reported in 41.7% of the sample, while alcohol consumption was 5.5% (Table 1). The most common cause of ESRD in our sample leading to HD was hypertensive nephropathy (29.6%), followed by diabetic nephropathy (16.1%). More than half complained of comorbidities (56.9%). Of the total sample, 81.9% had an AVF, and the rest had a CVC (Table 1).

**Table 1:** Sociodemographic characteristics of hemodialysis patients (*n* = 415) by type of vascular access (AVF vs. CVC).

Sociodemographic variable	Patients (*n* = 415)		*p*‐Value
	Arteriovenous fistula (*n* = 340)	Central venous catheter (*n* = 75)	
Age	62 (53–71)	63 (51–72)	0.916
Gender			
Male	205 (60.3%)	42 (56.0%)	0.517
Female	135 (39.7%)	33 (44.0%)	
Occupation			
Unemployed/retired	259 (76.2%)	63 (84.0%)	0.336
Self‐employed	32 (9.4%)	5 (6.7%)	
Employe	49 (14.4%)	7 (9.3%)	
Residency			
Akkar	1 (0.3%)	2 (2.7%)	0.003
Beirut	189 (55.6%)	33 (44.0%)	
Keserwan‐Jbeil	4 (1.2%)	1 (1.3%)	
Mount‐Lebanon	34 (10.0%)	14 (18.7%)	
Nabatieh	3 (0.9%)	0	
North	47 (13.8%)	3 (4.0%)	
South	62 (18.2)	22 (29.3%)	
Social status			
Single	62 (18.2%)	11 (14.7%)	0.891
Engaged	3 (0.9%)	1 (1.3%)	
Married	235 (69.1%)	54 (72.0%)	
Divorced	9 (2.6%)	3 (4.0%)	
Widowed	31 (9.1%)	6 (8.0%)	
Children			
Yes	259 (76.2%)	55 (73.3%)	0.656
No	81 (23.8%)	20 (26.7%)	
Relation with family			
Bad	2 (0.6%)	0	0.514
Neutral	45 (13.2%)	7 (9.3%)	
Good	293 (86.2%)	68 (90.7%)	
Educational level			0.519
Uneducated	152 (44.7%)	31 (41.3%)	
High school diploma	115 (33.8%)	24 (32.0%)	
University degree	69 (20.3%)	20 (26.7%)	
Other	4 (1.2%)	0	
Smoking			
Yes	148 (43.5%)	25 (33.3%)	0.121
No	192 (56.5%)	50 (66.7%)	
Alcohol			
Yes	17 (5.0%)	6 (8.0%)	0.277
No	323 (95.0%)	69 (92.0%)	

*Note:* A *p*‐value ≤ 0.05 is considered statistically significant.

### 3.2. Bivariate Analysis

Regarding pain, the median for the NRS scale was 2 (0–5) in both AVF and CVC groups. The median SF‐MPQ‐2 values for continuous pain, intermittent pain, neuropathic pain, and affective descriptors are reported in Table 2.

**Table 2:** Bivariate analysis of HADS, NRS, and SF‐MPQ‐2 between AVF and CVC (*n* = 415).

Scales	Patients (*n* = 415)		*p*‐Value
	Arteriovenous fistula (*n* = 340)	Central venous catheter (*n* = 75)	
	Median (IQR)	Median (IQR)	
HADS			
Anxiety	4 (2–8)	5 (2–10)	0.305
Depression	8 (3.25–12)	8 (4–14)	0.341
NRS	2 (0–5)	2 (0–5)	0.861
SFMPQ2			
Continuous	0 (0–1.167)	0 (0–1.33)	0.962
Intermittent	0 (0–0)	0 (0–0)	0.937
Neuropathic	0 (0–0.95)	0 (0–1)	0.644
Affective descriptors	0.17 (0–1.28)	(0–0.85)	0.173

*Note:* A *p*‐value ≤ 0.05 is considered statistically significant.

According to Table 3, most of our sample did not have anxiety (70.6% for AVF and 66.7% for CVC) nor depression (49.1% for AVF and 44.0% for CVC) in both VA groups. Abnormal anxiety (15.9% for AVF and 24% for CVC) and depression (34.4% for AVF and 36% for CVC) were more prevalent than borderline anxiety and depression in both groups.

**Table 3:** The prevalence of anxiety and depression among patients with different vascular access types.

Variable	Patients (*n* = 415)	
	Arteriovenous fistula (*n* = 340)	Central venous catheter (*n* = 75)
HADS‐anxiety		
Normal	240 (70.6%)	50 (66.7%)
Borderline	46 (13.5%)	7 (9.3%)
Abnormal	54 (15.9%)	18 (24.0%)
HADS‐depression		
Normal	167 (49.1%)	33 (44.0%)
Borderline	56 (16.5%)	15 (20.0%)
Abnormal	117 (34.4%)	27 (36.0%)

No statistical significance associated pain, anxiety, and depression with the type of VA running Mann Whitney *U* with *p* > 0.05 for HADS‐A, HADS‐D, NRS, and SFMPQ‐2 (Table 4).

**Table 4:** Bivariate analysis of secondary variables with HADS, NRS, and SF‐MPQ‐2 (*n* = 415).

Variables	HADS				NRS		SF‐MPQ‐2							
	Anxiety		Depression				Continuous		Intermittent		Neuropathic		Affective descriptor	
	Median (IQR)	*p*	Median (IQR)	*p*	Median (IQR)	*p*	Median (IQR)	*p*	Median (IQR)	*p*	Median (IQR)	*p*	Median (IQR)	*p*
Gender														
Male	4 (1–7)	<0.001	7 (3–12)	0.016	0 (0–4)	0.001	0 (0–1)	0.002	0 (0–0)	0.002	0 (0–0.6)	<0.001	0 (0–1)	0.013
Female	5 (2.25–11)		9 (4–14)		3 (0–5)		0.33 (0–2)		0 (0–0)		0 (0–1.35)		0.28 (0–1.53)	
Dialysis complication														
Yes	4 (2–9)	0.018	8 (4–13)	0.025	3 (0–5)	0.003	0.33 (0–1.5)	<0.001	0 (0–0)	0.138	0 (0–0.4)	0.013	0.28 (0–1.42)	<0.001
No	4 (1–7)		6 (2–12)		0 (0–3)		0 (0–0.5)		0 (0–0)		0 (0–1)		0 (0–0.71)	
Vascular access complication														
Yes	5 (2–9)	0.166	9 (4–13.25)	0.182	3 (0–6)	<0.001	0.833 (0–4.4)	<0.001	0 (0–1)	<0.001	0.1 (0–1.65)	0.003	0.78 (0–1.85)	<0.001
No	4 (2–8)		7 (3–13)		1 (0–4)		0 (0–1)		0 (0–0)		0 (0–0.8)		0 (0–1)	
Familiarization														
Yes	4 (2–8)	0.017	8 (3–12)	0.004	2 (0–5)	0.544	0 (0–1.33)	0.921	0 (0–0)	0.893	0 (0–1)	0.167	0.14 (0–1.28)	0.984
No	7 (3.25–11)		12.5 (4.25–16)		1 (0–4.75)		0.167 (0–0.125)		0 (0–0)		0 (0–0.65)		0.17 (0–1)	

*Note:* A *p*‐value ≤ 0.05 is considered statistically significant.

Females had higher levels of anxiety (26.2%) and depression (40.5%) than males (11.3% and 30.8%, respectively). As for pain, the median across all categories was null in males, with a slight elevation in females. Accordingly, gender differences were significant across all scales. Both dialysis and VA complications were higher in AVF (82.7% and 72.4%, respectively). Dialysis complications were associated with all scales used, except for intermittent pain. VA complications were significant with pain scales only; *p* < 0.001 for NRS, continuous pain, intermittent pain, and affective descriptors; and *p* = 0.003 for neuropathic pain. Regarding the time spent per the dialysis session, it was only associated with depression (*p* = 0.016), intermittent pain (*p* = 0.036), and affective descriptors of pain (*p* = 0.005). Adaptation to the VA was only significant with anxiety (*p* = 0.007) and depression (*p* = 0.004) (Table 4). Smokers on AVF reported higher rates of anxiety (51.8%) and depression (43%) compared to smokers on CVC (38.8% and 33.3%, respectively). Moreover, the prevalence of pain across the medical causes for dialysis was slightly higher in CVC (57.3%) than in AVF patients (53.8%).

### 3.3. Correlation Analysis

Pain, anxiety, and depression were positively correlated with each other. NRS was strongly correlated to continuous pain average (Spearman rho = 0.730, *p* < 0.001) and moderately correlated to neuropathic pain avg. (Spearman rho = 0.634, *p* < 0.001). Anxiety and depression were moderately correlated to each other (Spearman rho = 0.590, *p* < 0.001). Anxiety showed moderate correlation with both pain scales: NRS (Spearman rho = 0.403, *p* < 0.001) and continuous pain avg. (Spearman rho = 0.362, *p* < 0.001). Depression similarly showed moderate correlation with the same pain scales; NRS (Spearman rho = 0.384, *p* < 0.001) and continuous pain avg. (Spearman rho = 0.368, *p* < 0.001). Age was significantly correlated with depression in a positive manner (Spearman rho = 0.117, *p* = 0.017).

### 3.4. Multivariable Analysis

A multivariable analysis was conducted between the different variables in the study and the primary factors of pain, anxiety, and depression.

Of the factors studied for the association with HADS‐A, gender (adjusted *B*: −1.862; CI: −2.865–−0.860; *p*‐value<0.001), dialysis complication (adjusted *B*: −1.306, CI: −2.431–−0.181; *p*‐value = 0.023), and adaptation (adjusted *B*: 1.781; CI: 0.161−3.400; *p*‐value = 0.031) showed significance after adjustments (Table 5).

**Table 5:** Multivariable analysis of HADS‐anxiety across sociodemographic and dialysis‐related questions (*n* = 415).

Parameters	Univariate analysis			Multivariate analysis		
	*B*	Confidence interval	*p*‐Value	Adjusted *B*	Confidence interval	*p*‐Value
Age	0.006	−0.280 to 0.040	0.745	0.007	−0.029 to 0.042	0.708
Gender	−2.172	−3.138 to −1.206	<0.001	−1.862	−2.865 to −0.860	<0.001
Vascular access	−0.892	−2.150 to 0.336	0.164	−0.671	−1.913 to 0.572	0.289
Educational level	−0.184	−1.161 to 0.793	0.711	—	—	—
Social status	0.860	−0.201 to 1.922	0.112	0.483	−0.625 to 1.591	0.392
Time spent per dialysis session	0.664	−0.308 to 1.636	0.180	0.353	−0.615 to 1.322	0.474
Comorbidities	0.367	−0.612 to 1.346	0.416	—	—	—
Dialysis complications	−1.518	−2.655 to −0.381	0.009	−1.306	−2.431 to −0.181	0.023
Vascular access complication	−0.606	−1.747 to 0.535	0.297	—	—	—
Adaptation	1.851	0.217 to 3.485	0.026	1.781	0.161 to 3.400	0.031

*Note:* A *p*‐value ≤ 0.05 is considered statistically significant. (*R* ^2^ = 0.074; adjusted *R* ^2^ = 0.058).

Furthermore, studying the association of the same factors with HADS‐D, age (adjusted *B*: 0.0039; CI: 0.003–0.0076; *p*‐value = 0.036), dialysis complication (adjusted *B*: −1.280; CI: −2.480–−0.079; *p*‐value = 0.037), and adaptation (adjusted *B*: 2.905; CI: 1.179–4.632; *p*‐value = 0.001) showed significance after adjustments (Table 6).

**Table 6:** Multivariable analysis of HADS‐depression across sociodemographic and dialysis‐related questions (*n* = 415).

Parameters	Univariate analysis			Multivariate analysis		
	*B*	Confidence interval	*p*‐Value	Adjusted *B*	Confidence interval	*p*‐Value
Age	0.050	0.014 to 0.086	0.007	0.039	0.003–0.076	0.036
Gender	−1.390	−2.433 to −0.348	0.009	−1.007	−2.054 to 0.039	0.059
Vascular access	−0.672	−2.011 to 0.667	0.324	−0.382	−1.697 to 0.933	0.569
Educational level	0.720	−0.316 to 1.757	0.173	0.619	−0.399 to 1.637	0.233
Social status	0.048	−1.084 to 1.180	0.934	—	—	—
Time spent per dialysis session	1.430	0.403 to 2.456	0.006	0.990	−0.037 to 2.016	0.059
Comorbidities	−0.894	−1.931 to 0.144	0.091	−0.491	−1.538 to 0.557	0.358
Dialysis complications	−1.368	−2.579 to −0.156	0.027	−1.280	−2.480 to −0.079	0.037
Vascular access complication	−0.685	−1.898 to 0.528	0.268	—	—	—
Adaptation	2.806	1.080 to 4.533	0.002	2.905	1.179 to 4.632	0.001

*Note:* A *p*‐value ≤ 0.05 is considered statistically significant. (*R* ^2^ = 0.018; adjusted *R* ^2^ = 0.015).

Regarding pain, measured by the NRS and SF‐MPQ‐2, gender, dialysis, and VA complication showed association and were significant with *p*‐value<0.005 after adjustments. Age, VA, and comorbidities showed no significance with both scales (Tables 7 and 8).

**Table 7:** Multivariable analysis of SF‐MPQ‐2 across sociodemographic and dialysis‐related questions (*n* = 415).

Parameters	Univariate analysis			Multivariate analysis		
	*B*	Confidence interval	*p*‐Value	Adjusted *B*	Confidence interval	*p*‐Value
Age	0.020	−0.147 to 0.188	0.813	0.043	−0.124 to 0.209	0.613
Gender	−10.931	−15.688 to −6.173	<0.001	−8.021	−12.771 to −3.271	0.001
Vascular access	1.673	−4.542 to 7.888	0.597	3.305	−2.631 to 9.242	0.274
Educational level	5.079	0.286–9.872	0.038	2.850	−1.745 to 7.445	0.223
Social status	3.031	−2.212 to 8.274	0.256	—	—	—
Time spent per dialysis session	6.358	1.594–1.123	0.009	4.754	0.105–9.403	0.045
Comorbidities	4.192	−0.622 to 9.005	0.088	3.882	−0.856 to 8.620	0.108
Dialysis complications	−8.736	−14.325 to −3.147	0.002	−6.398	−11.847 to −0.948	0.022
Vascular access complication	−13.527	−19.006 to −8.049	<0.001	−12.217	−17.661 to −6.733	<0.001
Adaptation	0.859	−7.247 to 8.964	0.835	—	—	—

*Note:* A *p*‐value ≤ 0.05 is considered statistically significant. (*R* ^2^ = 0.126; adjusted *R* ^2^ = 0.109).

**Table 8:** Multivariable analysis of NRS across sociodemographic and dialysis‐related questions (*n* = 415).

Parameters	Univariate analysis			Multivariate analysis		
	*B*	Confidence interval	*p*‐Value	Adjusted *B*	Confidence interval	*p*‐Value
Age	0.006	−0.014 to 0.027	0.549	0.010	−0.011 to 0.032	0.342
Gender	−1.009	−1.606 to −0.412	0.001	−0.724	−1.330 to −0.119	0.019
Vascular access	0.017	−0.755 to 0.789	0.965	0.148	−0.610 to 0.907	0.701
Educational level	0.311	−0.286 to 0.909	0.306	—	—	—
Social status	0.204	−0.448 to 0.856	0.539	—	—	—
Time spent per dialysis session	0.522	−0.042 to 1.146	0.068	0.394	−0.200 to 0.988	0.193
Comorbidities	0.418	−0.181 to 1.016	0.171	0.455	−0.150 to 1.061	0.140
Dialysis complications	−1.033	−1.727 to −0.338	0.004	−0.872	−1.588 to −0.175	0.014
Vascular access complication	−1.157	−1.847 to −0.466	0.001	−1.027	−1.722 to −0.333	0.004
Adaptation	−0.379	−1.385 to 0.627	0.459	—	—	—

*Note:* A *p*‐value ≤ 0.05 is considered statistically significant. (*R* ^2^ = 0.071; adjusted *R* ^2^ = 0.055).

## 4. Discussion

The aim of this study was to compare the prevalence of pain, anxiety, and depression in chronic HD patients according to their type of VA (AVF vs. CVC). No significant difference was detected between AVF and CVC groups across the scales used, which prompts us to accept the null hypothesis. To better anchor our findings, we compared them to those found in the literature. The low pain outcome across categories with a null median in both VA groups is consistent with results from Masià‐Plana et al. [12] who reported low mean pain scores of 2.8 ± 1.9 in HD patients in general. No difference was noted between VA groups in relation to pain with *p* > 0.05 across all categories.

Likewise, anxiety (*p* = 0.305) and depression (*p* = 0.341) levels did not differ significantly between AVF and CVC groups in our study. However, several studies showed that there are higher anxiety and depression levels in patients with CVC [16–18]. Their studies did not take patients on dialysis for less than a year or those previously diagnosed with anxiety or depression into consideration. Different scales were used in our study than theirs (HADS vs. BAI/BDI/GDS, respectively); however, all these scales are considered comparable in terms of specificity and usage for screening for anxiety and depression [24].

Although our primary analysis did not reveal significant difference between pain, anxiety, or depression among VA groups, further data testing proved that dialysis related (*p* = 0.018) and VA related complications (*p* < 0.001) might be linked to these outcomes. Sikora et al. [27] also supported these findings by reporting that anxiety and depression are prevalent among dialysis patients due to their complications rather than the access type. Our exploratory analysis proved this point as participants with AVF had more dialysis‐ and VA‐related complications than those with CVC. The higher levels of depression observed in AVF participants can also be explained by this association.

There was a significant positive Spearman correlation between pain, anxiety, and depression scales. Semaan et al. [5] also found that anxiety and depression were significantly correlated (Spearman rho = 0.412, *p* < 0.001) in accordance with our findings. However, contrary to our study, they found only in one single center in Lebanon that anxiety and depression levels were high in dialysis patients in general.

In exploring the participants’ adaptation to their VA, they were asked whether they became more familiar with their VA and dialysis in general. Medians of anxiety levels (7 IQR 3.25–11) and depression levels (12.5 IQR 4.25–16) were higher in those who did not adapt to their VA. However, a fluctuating trend was observed when exploring the effect of the number of years on HD with the aspect of adaptation to the VA. Of the total sample, those who had been on HD for 1–3 years and had not adapted to their VA were 14.8%. This level decreased to 4.1% in those who had been on HD for 3–6 years and to 6.7% in those who had been on HD for more than 6 years. This might indicate that at the beginning of treatment, patients are not well‐accepting to the remodeling process or the insertion of an artificial venous access, but eventually, over time, they adapt better.

Upon running multivariable analysis, associations between the type of VA and the studied factors: pain, anxiety, and depression, remained insignificant (Tables 5–8). These regression results can be compared to those of Büberci et al. [16] who discussed their findings of the different levels of anxiety (96.8% for CVC vs. 67.2% for AVF, *p* < 0.001) and depression (90.3% for CVC vs. 52.9% for AVF, *p* < 0.001) between VA groups are not related to the VA itself, but rather to other attributes about the participants themselves. They suggested that their findings can be biased due to their low sample size (*n* = 101) collected from only one center.

Moreover, gender‐ and dialysis‐related complications remained significant after multivariable analysis with pain, anxiety, and depression (Tables 5–8). Taking the references into consideration with the adjusted *B* values, results prove that females scored an average of 1.862 points higher on the anxiety scale, 1.007 points higher on the depression scale, 8.021 points higher on the SF‐MPQ‐2 scale, and 0.724 points higher on the NRS scale. Our results partly agree with Masia Plana et al. who also found that females had more anxiety and depression than males [12]. Similarly, those who had dialysis complications scored higher on the anxiety scale by 1.306 points, on the depression scale by 1.280 points, and on the pain scales by 6.398 points (for SF‐MPQ‐2) and 0.872 points (for NRS), as previously discussed through the findings of Sikora et al. [27]. On the other hand, VA‐related complications were only significant with pain (Tables 5 and 6), indicating that those with an AVF had higher pain levels than those with CVC by 12.217 points (for SF‐MPQ‐2) and 1.027 points (for NRS). This is also in accordance with our bivariate analysis. Adaptation to the VA remained only significant with anxiety and depression in a positive way (Tables 5 and 6), meaning that patients who did not adapt to their VA scored higher on the anxiety scale by 1.781 points and on the depression scale by 2.905 points.

## 5. Limitations

Our study has several strength points. It is a multicentered study covering six hospitals across different regions of Lebanon with a large sample size of 415 patients. This gives it greater statistical power and reliability over smaller single‐centered studies. Our exclusion criteria minimize baseline psychiatric confounding factors by excluding patients who had taken antidepressants prior to initiating dialysis, who were diagnosed with anxiety and/or depression prior to their HD, and those who were on dialysis for less than a year.

Despite this, our study has several limitations. As this was a cross‐sectional study, establishing a direct relationship with psychosocial outcomes was restricted. Moreover, the levels of pain, anxiety, and depression were assessed through subjective reports from the participants, which rely on recall bias or differences in perception. In addition, sampling was done by double convenience for the hospitals as well as the participants.

## 6. Conclusion

In our multicenter study comprising 415 chronic HD patients, pain, anxiety, and depression did not differ significantly between types of VA. Instead, these outcomes were more closely linked to other variables studied such as time spent per dialysis session, dialysis and VA‐related complications, and adaptation to the VA. Our multivariable analysis further proved these results and may establish a framework for future research.

Taken together, our findings suggest that patient well‐being should not be framed primarily as a function of access type but as a consequence of symptom burden and preventable complications. It is necessary to implement routine screening in HD centers to evaluate patients’ psychological and physical conditions aside from their actual burden. Furthermore, future studies should focus not on evaluating the appropriate choice of VA but rather on exploring interventions that can provide proper psychological care to these patients.

## Funding

This project received no funding from any association or affiliation.

## Ethics Statement

This study was reviewed and approved by an independent institutional review board IRB. The study involved the analysis of fully anonymized patient data.

## Consent

As per the IRB consent form, the participants’ written and oral informed consent was collected prior to any data collection procedure.

## Conflicts of Interest

The authors declare no conflicts of interest.

## Data Availability

Datasets generated and analyzed during the current study are available from the corresponding author upon reasonable request.
